# Site-Specific Analysis of Inflammatory Markers in Discoid Lupus Erythematosus Skin

**DOI:** 10.1155/2014/925805

**Published:** 2014-03-11

**Authors:** Ryan B. Thorpe, Anna Gray, Kirthi R. Kumar, Joseph S. Susa, Benjamin F. Chong

**Affiliations:** ^1^Department of Dermatology, University of Texas Southwestern Medical Center, 5323 Harry Hines Boulevard, Dallas, TX 75390-9069, USA; ^2^Department of Pathology, University of Texas Southwestern Medical Center, Dallas, TX 75390-9072, USA; ^3^Cockerell Dermatopathology, Dallas, TX, USA

## Abstract

Prior studies identified T cells, B cells, and macrophages in the inflammatory infiltrate and up-regulation of their protein products in discoid lupus erythematosus (DLE) skin; however, they lacked rigorous analyses to define their specific locations in skin. Thus, we compared expressions of selected T cell, B cell, and macrophage markers in five areas of DLE, psoriasis, and normal skin. Immunostainings for CD3, CD4, CD8, CD20, CD68, CXCR3, CXCL10, and TIA-1 were performed in biopsies of 23 DLE lesional skin, 11 psoriasis lesional skin, and 5 normal skin. Three independent observers used a graded scale to rate each marker's presence in the epidermis, dermatoepidermal junction (DEJ), perivascular area, periadnexal area, and deep dermis. DLE lesional skin contained an increased abundance of CD3^+^, CD8^+^, and CD68^+^ cells at the DEJ, and CD20^+^ and CD68^+^ cells in the periadnexal area versus psoriasis and normal skin. CXCR3, CXCL10, and TIA-1 were elevated in periadnexal sites of DLE lesional skin versus psoriasis lesional skin. The aggregation of T cells, B cells, macrophages, and their protein products (CXCR3, CXCL10, and TIA-1) in the DEJ and periadnexal area of DLE lesional skin may contribute to the pathology of DLE through a coordinated, sophisticated process.

## 1. Introduction

As the most common manifestation of cutaneous lupus erythematosus [1, 2], discoid lupus erythematosus (DLE) is described as discrete, scaly erythematous papules and macules with peripheral hyperpigmentation most frequently found on the face, scalp, and neck [3, 4]. DLE lesions frequently expand during a period of active inflammation after which they heal with scarring, atrophy, and hyper- or hypopigmentation [4].

Histopathologically, inflammatory infiltrates consisting mainly of T cells, B cells, and macrophages [5] in DLE accumulate in perivascular and periadnexal areas and at the dermoepidermal junction (DEJ), resulting in vacuolar interface dermatitis [6]. Various studies have demonstrated that the predominant cells in DLE are T cells [6, 7] consisting of both CD4^+^ helper T cells and CD8^+^ cytotoxic T cells [5, 7–9]. In addition, DLE lesional skin exhibits significant amounts of protein products of CD8^+^ T cells including the T-cell restricted intracellular antigen 1 (TIA-1) and granzyme B [5, 6]. CXCR3, a chemokine receptor found on T_H_1 cells [10–12], and its ligands CXCL9 and CXCL10 have also been found to be up-regulated in DLE lesional skin [5]. These findings are corroborated by recent transcriptomic analyses in which DLE appears to be a T_H_1-mediated process [13]. Macrophages, which can be activated by T_H_1 cells, have been noted to be elevated at the DEJ and in the perivascular area in DLE lesional skin [5, 6, 14–16].

However, the literature has not been as consistent on the expression of B cells in DLE lesional skin [7, 17]. Investigators have observed a marked influx of B cells at the DEJ and in the perivascular area [6, 7, 18] and reported B cells to account for up to greater than 25% of the infiltrate in DLE lesional skin [19]. However, Tebbe et al. [20] and Lee et al. [21] noted that B cells were either rare or absent in the dermis of DLE lesional skin.

While describing where these cells are located in DLE lesional skin, these previous studies lacked site-specific comparisons with control skin that would determine which cells predominate at individual areas in DLE lesional skin. Identifying site predilection of these cells will provide greater insight into understanding the pathophysiology of DLE. Thus, we compared expression of T cell, B cell, and macrophage markers and their associated proteins, TIA-1, CXCR3, and CXCL10 in DLE lesional skin, psoriasis lesional skin, and normal skin. Psoriasis was chosen as a disease control because T cells and macrophages figure prominently in the pathology [22]. In addition to displaying T_H_17 and T_H_22 polarization, T cells in psoriasis also contain a sizable proportion of T_H_1 cells [23]. Because the dominant areas of inflammatory infiltrate in DLE lesional skin appear to be in the periadnexal area and dermoepidermal (DEJ) junction, we hypothesized that T cells, B cells, and macrophages accumulate in these two areas of DLE lesional skin when compared with psoriasis lesional skin and normal skin.

## 2. Materials and Methods 

### 2.1. Patient Recruitment

Biopsies of DLE lesional skin lesions were performed in the outpatient clinics of Parkland Memorial Hospital and University of Texas Southwestern Medical Center at Dallas, TX, from May 2004 to May 2011. The diagnosis of DLE was made by clinicopathological correlation. DLE patients were also evaluated for systemic lupus erythematosus (SLE) by determining the numbers of American College of Rheumatology SLE criteria satisfied [24, 25]. Medical charts for each DLE case were reviewed for demographic information, SLE criteria, and treatment at time of biopsy. Psoriasis lesional and normal skin biopsies were acquired from the archives of Cockerell Dermatopathology. Clinical information was unavailable for these biopsies. The institutional review board of the University of Texas Southwestern Medical Center approved this cross-sectional pilot study.

### 2.2. Immunohistochemistry Preparation

Formalin-fixed, paraffin-embedded tissue sections from each DLE, psoriasis, and normal skin biopsy specimen were cut into 4-micron thick sections and mounted on glass slides. Initial sections were stained with hematoxylin and eosin. Primary anti-human antibodies against CD3 (Cell Marque Corporation, Hot Springs, AR), CD4 (Biocare Medical, Concord, CA), CD8 (Cell Marque), CD20 (Ventana Medical Systems, Tucson, AZ), CD68 (Ventana), CXCL10 (R&D Systems, Minneapolis, MN), CXCR3 (BD Pharmingen, San Jose, CA), and TIA-1 (Biocare) were applied. Immunostaining using avidin-biotin-peroxidase complex and 3,3′-diaminobenzidine as substrate on an automated Ventana Benchmark instrument (Ventana) was performed using previously published protocols [26] with appropriately staining negative and positive control samples. Slides were counterstained with hematoxylin and bluing reagent.

### 2.3. Histopathology Analysis

Under basic light microscopy, three reviewers (AG, JS, BFC) examined the staining of each marker in DLE lesional skin, psoriasis lesional skin, and normal skin in five areas: epidermis, DEJ, perivascular area, periadnexal area, and deep dermis. Each area received a rating of 0 (none), 1 (mild), 2 (moderate), or 3 (high), which was based on a subjective assessment of the percentage of positively stained cells and the staining intensity.

### 2.4. Statistical Analysis

Since this was a pilot study, sample size was not calculated. Mean rating scores were compared amongst the three skin groups using the nonparametric Kruskal-Wallis Test and Dunn's Multiple Comparison Test using statistical software (GraphPad Prism 6 version 6.0c). Similar preplanned analyses were done between DLE subgroups (e.g., those with and without SLE). To reduce the number of false positives, given the numerous comparisons performed, *P* values of <0.01 were considered statistically significant.

## 3. Results and Discussion

### 3.1. Patient Characteristics

Twenty-three DLE patients were included in the study. The average age was 46.5 years. 91.3% and 60.9% of DLE patients were female and African Americans, respectively. DLE patients with SLE (DLE+/SLE+) were more likely to be on medications, such as hydroxychloroquine, mycophenolate mofetil, prednisone, and/or quinacrine, than DLE patients without SLE (DLE+/SLE−) (Table 1).

### 3.2. Site-Specific Immunostaining Analyses

**Figure 1:**
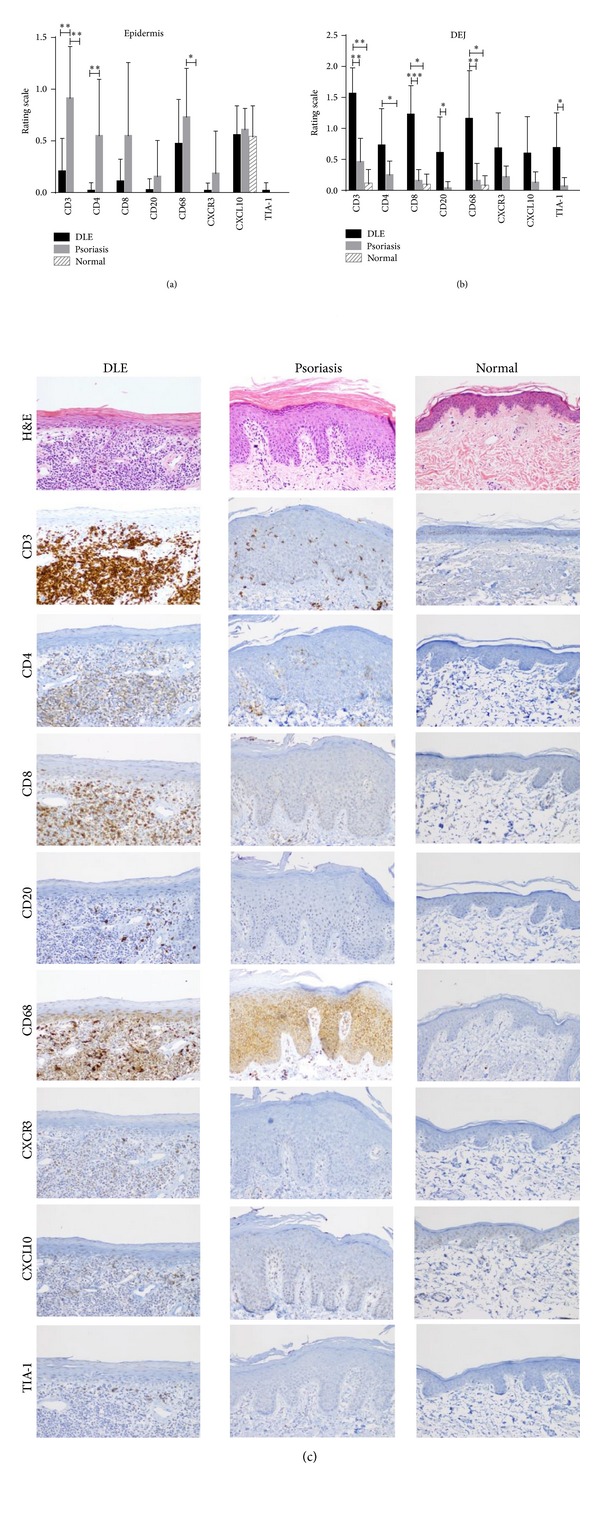
Comparisons of staining of T cell, B cell, and macrophage markers (CD3, CD4, CD8, CD20, CD68, CXCR3, CXCL10, and TIA-1) in the epidermis and dermoepidermal junction (DEJ) of DLE lesional skin (*N* = 23), psoriasis lesional skin (*N* = 11), and normal skin (*N* = 5). (a) The epidermis of psoriasis lesional skin contained higher levels of CD3^+^ and CD4^+^ T cells than DLE lesional skin. (b) The DEJ of DLE lesional skin expressed higher amounts of CD3^+^ T cells, CD8^+^ T cells, and CD68^+^ macrophages than psoriasis lesional skin and normal skin and higher amounts of CD4^+^ T cells, CD20^+^ B cells, and TIA-1 versus at least one of the control populations studied. *P < 0.01; **P < 0.001; ***P < 0.0001. (c) H&E and various immunostainings showed that the epidermis of psoriasis lesional skin contained higher levels of CD3^+^ and CD4^+^ T cells, while the DEJ of DLE lesional skin expressed higher amounts of CD3^+^ T cells, CD8^+^ T cells, and CD68^+^ macrophages. Magnification: 100x.

**Figure 2:**
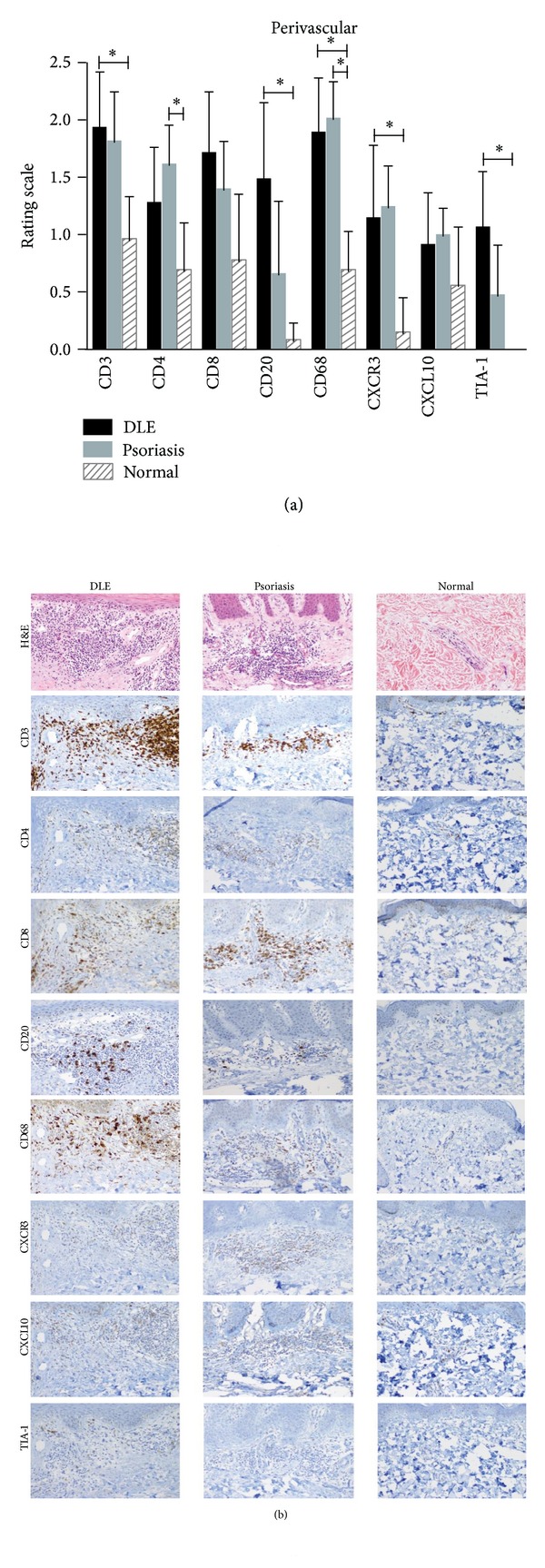
Comparisons of staining of T cell, B cell, and macrophage markers (CD3, CD4, CD8, CD20, CD68, CXCR3, CXCL10, and TIA-1) in the perivascular area of DLE lesional skin (*N* = 23), psoriasis lesional skin (*N* = 11), and normal skin (*N* = 5). (a) The perivascular area of DLE lesional skin contained higher levels of CD3^+^ T cells, CD20^+^ B cells, and CD68^+^ macrophages, CXCR3, and TIA-1 compared with normal skin. *P < 0.01; **P < 0.001; ***P < 0.0001. (b) H&E and various immunostainings showed that the perivascular area of DLE lesional skin contained higher levels of CD3^+^ T cells, CD20^+^ B cells, and CD68^+^ macrophages, CXCR3, and TIA-1 compared with normal skin. No significant differences were discovered between DLE and psoriasis lesional skin. Magnification: 100x.

**Figure 3:**
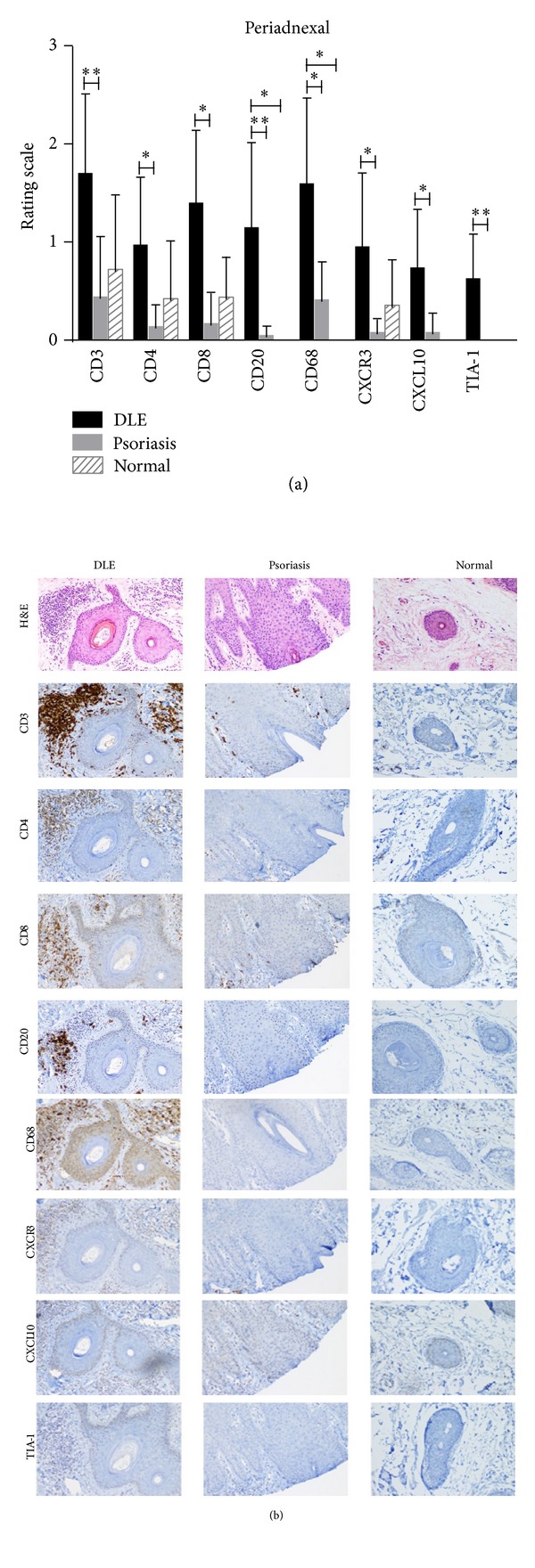
Comparisons of staining of T cell, B cell, and macrophage markers (CD3, CD4, CD8, CD20, CD68, CXCR3, CXCL10, and TIA-1) in the periadnexal area of DLE lesional skin (*N* = 23), psoriasis lesional skin (*N* = 11), and normal skin (*N* = 5). (a) DLE lesional skin expressed higher levels of all the markers studied in the periadnexal area compared with psoriasis lesional skin. *P < 0.01; **P < 0.001; ***P < 0.0001.(b) H&E and various immunostainings showed that DLE lesional skin expressed higher levels of all the markers studied in the periadnexal area compared with psoriasis lesional skin. Magnification: 100x.

**Figure 4:**
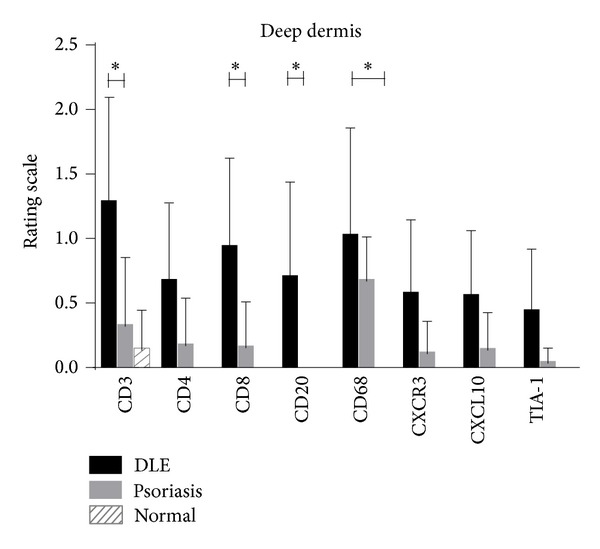
Comparisons of staining of T cell, B cell, and macrophage markers (CD3, CD4, CD8, CD20, CD68, CXCR3, CXCL10, and TIA-1) in the deep dermis of DLE lesional skin (*N* = 23), psoriasis lesional skin (*N* = 11), and normal skin (*N* = 5). DLE lesional skin expressed higher amounts of CD3^+^ T cells, CD8^+^ T cells, CD20^+^ B cells, and CD68^+^ cells than at least one of the control populations studied. *P < 0.01; **P < 0.001; ***P < 0.0001.

In the epidermis, psoriasis lesional skin (*N* = 11) was stained more for the T cell markers, CD3, and CD4, than DLE lesional skin (*N* = 23). No major differences were seen between DLE lesional and normal skin epidermis (*N* = 5) (Figure 1). At the DEJ, CD3^+^ T cells, CD8^+^ cytotoxic T cells, CD20^+^ B cells, CD68^+^ macrophages, and TIA-1 were all higher in DLE lesional skin than in psoriasis lesional skin. When comparing DLE with normal skin at the DEJ, similar findings were found except that CD20^+^ B cells and TIA-1 were not significantly different, and CD4^+^ helper T cells were significantly higher in DLE lesional skin (Figure 1). In the perivascular area, no significant differences were discovered in DLE lesional skin versus psoriasis lesional skin. Conversely, when compared with normal skin, DLE lesional skin demonstrated significantly higher staining for CD3, CD20, CD68, CXCR3, and TIA-1 in the perivascular area (Figure 2). Of all five areas analyzed, the periadnexal area showcased the most marked dissimilarities between DLE and psoriasis lesional skin. All markers studied were expressed at significantly higher levels in DLE than in psoriasis lesional skin at this site. Fewer differences were noted when comparing DLE with normal skin, with only CD20 and CD68 being up-regulated in the periadnexal areas of DLE lesional skin (Figure 3). Lastly, DLE lesional skin exhibited significantly more staining than psoriasis lesional skin for CD3, CD8, and CD20 in the deep dermis. Only CD68^+^ macrophages significantly populated the deep dermis of DLE lesional skin versus normal skin (Figure 4).

**Figure 5:**
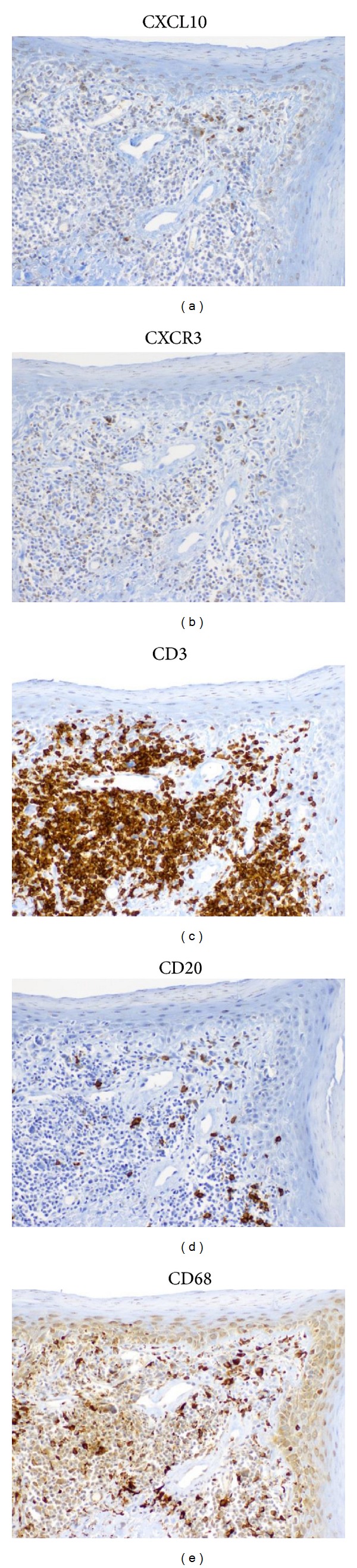
Serial sections of DLE lesional skin showed that CXCL10 (a) and CXCR3 (b) appeared to mirror the staining patterns of CD3^+^ T cells (c) and CD68^+^ (d) macrophages, but not CD20^+^ (e) B cells. Serial sections of DLE lesional skin were stained for CD3, CD20, CD68, CXCL10, and CXCR3. Magnification: 200x.

Serial sections revealed that CXCR3 and CXCL10 appeared to have similar staining patterns as the CD3^+^ T cell and CD68^+^ macrophage populations, but not the CD20^+^ B cell populations (Figure 5). Immunostainings of the markers studied were also compared in the skin of DLE+/SLE− with DLE+/SLE+ patients. While the data trended towards increased staining in DLE+/SLE− skin for all of the markers studied, none were found to be significantly different in any of the five locations studied (data not shown).

## 4. Discussion

We analyzed the T cell, B cell, and macrophage populations and selected associated proteins (CXCR3, CXCL10, and TIA-1) in five different areas of DLE lesional skin. To determine where these markers were differentially expressed in DLE lesional skin; site-specific comparisons were made with psoriasis lesional skin and normal skin. We stratified our results into major or minor categories based on whether DLE was significantly discrepant from psoriasis and normal skin (major) or from only one of the two control groups (minor).

A major finding of T cells in DLE lesional skin was that CD3^+^ T cells and CD8^+^ cytotoxic T cells were significantly higher in DLE lesional skin versus both psoriasis lesional skin and normal skin at the DEJ. Minor findings included increased numbers of CD3^+^ T cells and CD8^+^ T cells in the periadnexal area and in the deep dermis in DLE versus psoriasis lesional skin and CD4^+^ helper T cells at the DEJ and in periadnexal areas in DLE lesional skin versus normal and psoriasis lesional skin, respectively. Although Tebbe et al. reported that most T cells were of the helper subtype [20], our results highlight an elevation of CD8^+^ cytotoxic T cells in DLE lesional skin, particularly at the DEJ, compared with their psoriasis and normal counterparts. A byproduct of cytotoxic T cell activity is TIA-1, which is found in azurophilic cytoplasmic granules [27]. TIA-1 is an intracellular membrane-associated protein that induces DNA fragmentation, resulting in apoptosis [28]. DLE lesional skin also demonstrated increased TIA-1 at the DEJ and in the periadnexal areas, which are both near keratinocytes and areas where CD8^+^ T cells were also elevated. Thus, it is likely that TIA-1 released by CD8^+^ T cells is associated with cellular damage resulting in hydropic degeneration and apoptosis of keratinocytes located in the epidermis and periadnexal areas. Furthermore, these apoptotic keratinocytes become likely sources of nuclear material that could be targets of autoantibodies.

B cells have largely been implicated in SLE due to their production of autoantibodies. In SLE, autoantibodies can form immune complexes that mediate tissue damage through antibody-dependent cell-mediated cytotoxicity (ADCC) [29]. Moreover,* in vitro* experiments involving UVB-irradiated keratinocytes isolated from SLE or subacute cutaneous lupus skin showed increased binding of anti-Ro IgG antibodies, resulting in enhanced ADCC. In contrast, irradiated keratinocytes from DLE patients did not show enhanced ADCC [30, 31]. Despite these negative findings in DLE lesional skin, our data showing B cells congregating in different areas of DLE lesional skin supports the notion of B cells and potentially autoantibodies being involved in the pathology of DLE. The major result was that B cells in DLE lesional skin were significantly higher in periadnexal areas versus those from both control groups. We postulate that autoantibodies produced by these B cells target nearby apoptotic keratinocytes displaying nuclear antigens at their cellular surface. B cells were also increased in DLE lesional skin at the DEJ, perivascular area, and in the deep dermis when compared with one of the control groups. As our data has indicated multiple areas in DLE lesional skin with significant involvement of B cells, it contrasts the findings of Tebbe et al. [20] and Lee et al. [21] that did not find enhanced levels of B cells in DLE lesional skin. One plausible explanation for the discrepancy in the literature is the methods used in the various analyses. By parceling DLE lesional skin into specific locations and monitoring for B cells at each location as opposed to analyzing the entire skin at one time, we were able to perform site-specific analyses to confirm the accumulation of B cells in various sites of DLE lesional skin.

Similar to CD8^+^ T cells, CD68^+^ macrophages were also significantly increased at the DEJ in DLE lesional skin versus both psoriasis lesional skin and normal skin. Additionally, significantly higher levels of CD68^+^ macrophages were found in the periadnexal area of DLE lesional skin versus both control groups. Of all the cell types, the CD68^+^ macrophage was the only cell type that was consistently significantly different when compared with normal skin in 4 of 5 locations studied (DEJ, perivascular area, periadnexal area, and deep dermis). Despite the enhanced presence of macrophages in DLE lesional skin, little is known regarding how macrophages contribute to the pathology of DLE. Previous studies have found that after CD3^+^ T cells, CD68^+^ cells are the next most common infiltrate in DLE [5, 6]. CD68^+^ macrophages may be recruited to DLE lesional skin, specifically to the DEJ and periadnexal sites, to engulf apoptotic keratinocytes. This phagocytosis could be mediated through ADCC, as macrophages can target apoptotic cells marked by autoantibodies [32]. However, it is unclear whether their accumulation is due to defective clearance of these keratinocytes or increased keratinocyte apoptosis [33]. In addition to its phagocytic properties, macrophages can highly influence T cell differentiation by secreting cytokines such as IL-12 that favor a T_H_1 bias. IL-12 can favor differentiation of naïve T cells to T_H_1 cells and promote greater IFN-*γ* production by T_H_1 cells [34]. Further inquiries are required to learn more about the specific makeup, character, and regulation of macrophages in DLE.

CXCL10 and CXCR3, which were both up-regulated in the periadnexal areas of DLE versus psoriasis lesional skin, appeared to mimic the expression patterns of effector T cells and macrophages. As CXCR3 is predominantly found on T_H_1 and cytotoxic T cells [35, 36], we have confirmed this finding on DLE lesional skin, as CXCR3 stainings have paralleled those of CD3^+^ and CD8^+^ T cells. Furthermore, Wenzel et al. [5] reported that CXCR3 was found in 60–90% of lymphocytes in lesional cutaneous lupus skin. Additionally, they also noted CXCL10 staining in selected areas of the basal epidermis containing interface dermatitis of DLE lesional skin [5]. Along with the other CXCR3 ligands, CXCL9 and CXCL11, CXCL10 is richly expressed in cutaneous lupus skin [37, 38]. As activated macrophages produce CXCL10, which can help recruit T cells [39], macrophages in DLE are likely a major source of CXCL10. Thus, we propose that macrophages drive the recruitment of lymphocytes through production of CXCL10, particularly in the periadnexal areas of DLE lesional skin. Further double immunofluorescence or dual chromagen immunohistochemistry studies will be planned to corroborate these findings.

We also investigated if there were differences in the inflammatory infiltrates in DLE+/SLE− and DLE+/SLE+ skin because it is unclear whether DLE in the setting of systemic disease is different than DLE alone. While no significant differences in any marker between DLE+/SLE− and DLE+/SLE+ skin were found, there were trends towards stronger immunostaining of CD3, CD4, CD8, CD20, CD68, CXCR3, CXCL10, and TIA-1 in most areas of DLE+/SLE− skin compared with DLE+/SLE+ skin. We hypothesize that a dilutional effect may be present in DLE patients with systemic disease in which inflammatory cells and their protein products are dispersed in other areas of the body. However, medication effect may also explain this difference, as more DLE+/SLE+ patients were on potent immunosuppressants than DLE+/SLE− patients.

The study is limited by the reviewers not being blinded during the immunohistochemical analyses. While reviewers were instructed to focus their attention on the staining intensities of the various markers and not on the underlying pathology, distinct pathological findings in DLE, psoriasis, and normal skin could be viewed in the background. Additionally, clinical data was not available for the control groups. While no significant differences in the markers studied were seen between DLE+/SLE+ and DLE+/SLE− skin, we are pursuing further investigations in these DLE skin subtypes that broaden the number of genes and proteins studied via gene microarrays and confirmatory protein expression studies.

## 5. Conclusions

In conclusion, we performed site-specific analyses of the inflammatory infiltrate and their selected proteins in DLE lesional skin compared with psoriasis lesional skin and normal skin. We found that CD3^+^ T cells, CD8^+^ T cells, and CD68^+^ macrophages are significantly higher in the DEJ of DLE lesional skin versus normal and psoriasis lesional skin. CD20^+^ B cells and CD68^+^ macrophages were also elevated in the periadnexal areas of DLE lesional skin compared with normal and psoriasis lesional skin. These periadnexal areas also contained higher expression of TIA-1, CXCR3, and CXCL10 in DLE versus psoriasis lesional skin. Aggregation of these cells in these specific areas of DLE lesional skin likely contributes to the pathology of DLE through an intricate cross-talk and coordination amongst these cells.

## Figures and Tables

**Table 1:** DLE patient characteristics.

Patient no.	SLE (Y/N)	Age	Gender	Ethnicity	No. of SLE Criteria	Treatment at biopsy	Biopsy site
1	N	55	F	Caucasian	3	—	Shoulder
2	N	52	F	Caucasian	3	HCQ	Scalp
3	N	46	F	Caucasian	3	—	Scalp
4	N	41	F	AA	1	—	Scalp
5	N	50	F	AA	2	—	Chin
6	N	42	F	Caucasian	1	—	Cheek
7	N	47	M	AA	2	—	Scalp
8	N	31	F	AA	1	—	Scalp
9	N	36	F	AA	3	—	Scalp
10	N	62	F	Caucasian	3	—	Cheek
11	N	45	F	Caucasian	2	—	Nasal Ala
12	N	41	F	AA	3	—	Scalp
13	N	50	M	AA	2	—	Scalp
14	Y	51	F	Asian	5	HCQ, MM, prednisone	Scalp
15	Y	42	F	Hispanic	6	Prednisone	Arm
16	Y	31	F	AA	9	MM, prednisone, quinacrine	Upper thigh
17	Y	39	F	AA	5	HCQ, prednisone	Scalp
18	Y	73	F	Caucasian	7	Prednisone	Cheek
19	Y	54	F	AA	5	HCQ	Shoulder
20	Y	54	F	AA	4	—	Scalp
21	Y	36	F	AA	5	Prednisone	Scalp
22	Y	42	F	AA	6	—	Scalp
23	Y	50	F	AA	5	—	Shoulder

Abbreviations: AA: African American, DLE: discoid lupus erythematosus, HCQ: hydroxychloroquine, MM: mycophenolate mofetil, SLE: systemic lupus erythematosus.

## Abbreviations

ADCC: Antibody-dependent cell-mediated cytotoxicity
DEJ: Dermoepidermal junction
DLE: Discoid lupus erythematosus
SLE: Systemic lupus erythematosus
TIA-1: T-cell restricted intracellular antigen 1.
